# Fibrotic Diseases of the Human Urinary and Genital Tract: Current Understanding and Potential Strategies for Treatment

**DOI:** 10.3390/jcm12144770

**Published:** 2023-07-19

**Authors:** Harrina E. Rahardjo, Viktoria Märker, Dimitrios Tsikas, Markus A. Kuczyk, Stefan Ückert, Andreas Bannowsky

**Affiliations:** 1 Department of Urology, Cipto Mangunkusumo Hospital, School of Medicine, University of Indonesia, Jakarta 10430, Indonesia; harrinaerlianti@gmail.com; 2 Department of Urology & Urological Oncology, Division of Surgery, Hannover Medical School, 30625 Hannover, Germany; kuczyk.markus@mh-hannover.de (M.A.K.); streetgang@gmx.de (S.Ü.); 3 Department of Forensic Psychiatry, University Hospital Hamburg-Eppendorf (UKE), 20251 Hamburg, Germany; v.maerker@uke.de; 4 Core Unit Proteomics, Center of Pharmacology & Toxicology, Hannover Medical School, 30625 Hannover, Germany; 5 Department of Urology, Imland Hospital gGmbH, 24768 Rendsburg, Germany; andreas.bannowsky@imland.de

**Keywords:** urology, fibrotic diseases, pathophysiology, treatment strategies

## Abstract

Fibrosis is a disease condition characterized by abnormalities of the extracellular matrix, such as accumulation of the transforming growth factor β, infiltration by myofibroblasts, deposition of collagen, and a generalized dysregulation of collagen maturation. It can severely impair the function of organs by replacing normal tissue with a highly collagenized matrix, thereby reducing the elasticity and compliance of tissues. Fibrotic diseases of the genitourinary tract present relevant problems in healthcare, and their principles of pathophysiology remain unclarified; hence, the armamentarium for prevention and treatment is limited. These diseases include renal fibrosis, Peyronie’s disease and ureteral and urethral strictures due to perturbations in the process of wound healing in response to injuries. Such deteriorations may contribute to obstructive uropathies or sexual dysfunction. This review provides a brief overview of the most frequent fibrotic diseases of the genitourinary system and of how the pathophysiology is related to symptoms, and also highlights potential therapeutic strategies to address the abnormal deposition of collagen. Although the understanding of factors associated with fibrotic conditions of the urinary and genital tract is still limited, some beneficial advances have been made. Further research will serve to provide a more comprehensive insight into factors responsible for the development of fibrotic tissue deposition.

## 1. General Mechanisms in the Pathophysiology of Fibrotic Diseases

Tissue fibrosis is a physiological process in which cellular homeostasis is disrupted and extracellular matrix is excessively deposited. This condition can affect several organs or local tissue entities of the human body, such as the cardiovascular and pulmonary tract, the digestive system (intestine, liver), peritoneum, skin, and also the upper and lower urinary tract and genital structures (for example, the penis). All fibrotic alterations may finally lead to vital failure in organ or tissue function, and there is no effective medication or therapy available to treat this condition. In general, fibrotic diseases are characterized by inflammatory responses resulting in the production and accumulation of extracellular matrix (ECM) components (collagen fibers, fibronectin, proteoglycans, and integrins, all of which may enhance further the activation of pro-fibrotic mediators, such as the transforming growth factor β1), as well as the proliferation, migration and accumulation of mesenchymal cells. Migration of macrophages, T-cells and granulocytes to different tissues is also a distinctive feature in these diseases, along with an increased production of pro-inflammatory mediators leading to an increase in the number of fibroblasts in the local area. Intermittent phases of progression, acute exacerbation and remission can be found in fibrotic diseases; the causes of these diseases are distinctive, as they target different organs [1]. In the human urogenital tract, inflammatory mediators can trigger the proliferation of resident fibroblasts or the transformation of endothelial or epithelial cells into myofibroblasts. In cases where epithelial or endothelial cells are transformed, a loss in apical-basal polarity and adhesion properties occurs, together with a down-regulation of epithelial markers (such as E-cadherin and occludin) and an up-regulation of mesenchymal markers, such as smooth muscle alpha-actin, vimentin, fibronectin and fibroblast-specific protein 1 (FSP 1). As a result of this phenotypic process, the cells acquire mesenchymal features, including the ability to migrate and infiltrate. These myofibroblasts share phenotypic properties of both fibroblasts and smooth muscle cells and play a critical role in collagen deposition and preventing normal functional tissue from being rebuilt. The abnormal persistence of these cells leads to hypertrophic formation and other fibrotic conditions. Inflammation, hypoxia and senescence are three conditions closely associated with fibrogenesis and the damage caused to the tissue by scarring. These issues interact and converge at the pleiotropic cytokine transforming growth factor β (TGF β). TGF β has an important function in tissue inflammation, fibrosis, cell apoptosis, and proliferation, and is mostly known for its role as the main pro-fibrotic mediator [1,2]. At least three isoforms of TGF β are found in mammals: TGF β1, β2 and β3, whereas ß1 is more abundantly produced. In response to an endogenous stimuli, such as exposure to reactive oxygen species conditions, oxidative stress (low oxygen tension in the tissue), or pro-inflammatory cytokines, such as tumor necrosis factor-α (TNF-α) or interleukin 6 (IL-6), immune cells are activated to generate TGF β1. Once released and activated, TGF β1 binds to the type I and type II TGF β receptors (TβRI and II), finally leading to the phosphorylation of a receptor-associated suppressor of Smad proteins (R-Smads, in particular Smad 1 and 3), known to trigger the transcription of genes related to pro-fibrotic proteins, and also suppressing the transcription of anti-fibrotic mRNAs [3]. Persistent inflammation exacerbates this cascade, thereby stimulating fibrogenesis, causing further excessive accumulation of ECM and inhibiting the activity of collagenases and proteases, enzymes known to counteract ECM remodeling. In turn, the generation and release of TGF β can be regulated reversely by activating the so-called mitogen-activated protein kinase (MAPK), thereby counteracting the nuclear translocation and binding of Smad to the DNA. Thus, by inhibiting Smad1, MAPK can antagonize the entire TGF β response [4]. To date, it is widely accepted that the dysregulation in the generation of TGF ß1 may explain the pathophysiological mechanism underlying the manifestation of fibrotic diseases. The crucial role of TGF ß1 was convincingly identified in Marfan Syndrome (MFS, also known as dolichostenomelia), a congenital malformation syndrome of autosomal dominant inheritance, affecting the connective tissue. This condition is rare (approximately 1 in 3000 to 5000 people) but is a severe disease that is very difficult to treat. Due to a mutation in FBN1, a gene located on chromosome 15 and encoding for fibrillin 1, a macromolecule that polymerizes to microfilaments and connects to elastin to form elastic fibers of the extracellular matrix, a structural abnormality of elastic fibers arises. Fibrillin 1 also has a regulatory role in TGF ß1 signaling [5,6]. Usually, patients with MFS are phenotypically characterized by multiple joint contractures, thoracolumbar kyphoscoliosis, long, spider-like limbs, fingers and toes, and exhibit an increased serum level of TGF ß1 [7,8]. The pronounced increase in TGF ß1 signaling leads to the excessive production of collagen and also upregulates the levels of elastase and matrix metalloproteinases. This finally results in the degradation of elastic fibers, thereby causing potentially life-threatening cardiovascular manifestations, such as congestive heart failure, aortic root aneurism, acute aortic dissection (AAD) and/or mitral valve prolapse, known as the most severe deteriorations of MFS. MFS is a pleiotropic disease, so that a single mutation can cause multiple symptoms, and not all patients present with the same symptoms. Interestingly, it has been shown that TGF ß1 decreases with successful treatment of, for example, the cardiovascular (aortic) conditions of the disease with antagonists of calcium channels, ß-adrenergic or angiotensin receptors or compounds inhibiting the angiotensin-converting enzyme (ACE) [9]. Chiefly, the latter have been proposed as a reasonable therapeutic option, since it has been investigated that the endogenous peptide angiotensin II (AT II) acts as a vasoactive mediator in the RAS by activating AT receptors, thus contributing to fibrotic transition and the deposition of ECM in responses to inflammation or excess angiogenesis/cell proliferation. Given the fact that numerous AT II inhibitory drugs are available on the market, it remains to be proven whether the application of an AT receptor blocker would enable the decrease in TGF β and, hence, prevention of fibrosis formation [10]. Up until now, urogenital malfunctions associated with MFS have not been studied in detail. Although not proven, it seems likely that urinary incontinence and other voiding dysfunctions may occur at an early stage and to a severe degree in both women and men affected by the malady. However, it has been indicated that chronic lower urinary tract symptoms in patients with MS are due mainly to complications following aortic surgery or are caused by the compression of sacral nerve roots [11,12].

## 2. Renal Fibrosis

In the kidneys, hypertension, diabetes, ischemia, oxidative stress, inflammation or urinary tract obstruction with increased hydrostatic force can cause trauma to the renal tissue, resulting in the elevation of TGF β1, thereby initiating the synthesis of ECM and hence fibrotic remodeling of the tissue [13]. It has been reported that from 10% up to almost 30% of myofibroblasts found in renal fibrosis originated from tubular epithelial cells (TEC) and also endothelial cells [14,15] Apart from the TECs, podocytes have also been identified as progenitor cells in the pathogenesis of renal fibrosis. For example, it has been demonstrated that diabetic nephropathy, induced by high glucose levels, is correlated with the transcription of fibronectin, the activation of TGF β1 and the mesenchymal transformation of podocytes [16,17]. However, the exact proportion of cells that transform into myofibroblasts is still unclear, since the data provided are inconsistent. In addition, there is some evidence that a significant number of said cells express both epithelial and mesenchymal markers and contribute to the onset of fibrosis, not by differentiating into fibroblasts but due to a loss of functionality, thus impairing epithelial regeneration and affecting the inflammatory milieu [18,19]. This conception, designated as partial epi-/endothelial-mesenchymal transformation (pEMT), may offer novel perspectives on the pathogenesis and treatment options for renal fibrosis/diabetic nephropathy, which should also take into account the targeting of podocytes. The proliferation of fibroblasts is mainly promoted by the inflammatory process. According to results from animal models, there are four major steps leading to renal fibrosis: the production of cytokines, growth factors and TGF β1, caused by macrophages on the interstitial level, stimulates the proliferation of fibroblasts (characterized by the expression of smooth muscle alpha-actin and other mesenchymal markers proteins), tubular apoptosis, and hence the necrosis of tubular cells, which results finally in tubular atrophy. Other factors, such as oxygen-free radicals produced by exogenous or endogenous compounds, and biologically active peptides (for example, the epidermal and insulin-like growth factor) might also play a relevant role [20,21]. There are hints that the effects of TGF β1 in the formation of renal fibrosis are also mediated via the upregulation of microRNAs (miRNAs). miRNAs play a role in various biological processes, and it has been shown that several miRNAs may promote the formation of fibrosis. In particular, miR-21 has been well investigated; it is transcribed in response to the release of TGF β1 and the interaction of the growth factor with Smad 3. On the contrary, Smad 2 counter-regulates the activity of Smad 3, and has thus potentially been associated with an anti-fibrotic, protective signaling pathway in the kidney [22,23]. In the light of the role of miR-21 in kidney fibrosis, the use of antisense oligonucleotides targeting this particular microRNA has been proposed as a potential therapeutic strategy. In fact, the suppression of miR-21 still poses significant challenges, particularly in terms of targeting the treatment to the target tissue and adjusting the optimal drug concentration to ensure effectiveness and minimize side effects [24]. Recently, RG-012 (Sanofi-Genzyme, Clinical Development, Cambridge, MA, Eloxx Pharmaceuticals, Watertown, MA, USA), a powerful inhibitor of miR-21, has been undergoing clinical assessment to evaluate its safety, tolerability and pharmacokinetics in healthy subjects [25].

## 3. Ureteral Fibrosis

As outlined above, TGF β1 remains the main culprit in the deposition of ECM and hence the production of fibrotic tissue. In the ureter, the formation of fibrotic tissue mediated by TGF β1 may cause strictures which eventually lead to (unilateral) ureteral obstruction (UUO). This chain of events starts with disturbances in the healing of wounds brought about by traumatic lesions and/or inflammation, later triggering the expression of inflammatory cytokines and the onset of epithelial/endothelial-mesenchymal transformation [26]. The deposition of collagen and events of scarring in the strictures are closely related to an increased expression of TGF β1. Due to changes in hydrostatic pressure and an increase in oxidative stress, the persisting obstruction may cause damage to the kidney. Acute kidney infections that can worsen to a chronic state are also frequently seen. In the chronic state, the constant production of collagen by fibroblasts is the main contributor to the development of severe deteriorations in kidney function [27,28]. Up until now, no standardized pharmacological treatment of ureteral fibrosis has been established. Some treatment approaches include the application of vitamins (for example, amygdalin, also known as vitamin B17) and antioxidants (alpha-lipoic acid and the natural, plant-derived compounds curcumin and thymoquinone), as well as recombinant polypeptides or small interfering (si) RNAs. Recent research has shown that drugs targeting the positive feedback loop between the renin–angiotensin–aldosterone system (RAS) and inflammation may have the potential to delay the process of fibrosis. Thus, a proposed strategy to target ureteral fibrosis is to block the action of RAS by antioxidants such as vitamins, alpha-lipoic acid (a cofactor of several mitochondrial dehydrogenases), curcumin and thymoquinone, or aliskiren (a drug acting as an inhibitor of RAS and renin), resulting in a decrease in the inflammatory process (by inhibiting the production of fibrotic and inflammatory proteins) and enabling the prevention of oxidative stress. Antagonizing the expression of angiotensin II (AT II) by interfering with the NF-kB pathway has been identified as another potential option for treatment [29].

## 4. Urethral Fibrosis

Regarding urethral fibrosis, several studies have been conducted in order to describe in detail the pathways leading from local inflammation, the release of cytokines, including TGF β1, to the activation of fibroblasts, the excess deposition of fibrotic tissue and the manifestation of strictures, promoted by the accumulation of dense collagen fibers lacking elasticity [30]. A major contribution of miRNA to the process leading to the formation of ECM in urethral fibrosis has been implicated; however, the underlying molecular mechanism are still under debate. In patients with the so-called pelvic fracture urethral distraction defect (PFUDD), five different types of miRNAs (designated as has-miR-129-5p, has-miR-135a-5p, msa-miR-363-3p, has-miR-6720-3p and has-miR-9-5p) were identified that could be possibly related to the pathway of fibrogenesis mediated by TGF β1. In addition, there is some evidence that, in particular, mo-miR-339-5p and mo-miR-31a-3p, known to bind to the tumor suppressor genes *Tp53inp* and *Dab2ip*, respectively, may also play a role in the deposition of fibrin, the migration of urethral epithelial cells, and urethral tissue remodeling. In the rat model, it was demonstrated that, in response to provoked urethral injuries, the transcription of some miRNAs were markedly up-regulated (rno-miR-212-5p, rno-miR-31a-3p, rno-miR-34b-3p, rno-miR-532-3p, and rno-miR-31a-5p), whereas others were down-regulated (rno-miR-486, rno-miR-503-5p, rno-miR-376a-3p, and rno-miR-410-3p) [31,32]. It remains to be elucidated whether this will provide a basis for future potential therapies to treat urethral fibrosis. Other signaling cascades, involving the Smad and MAPK system, have also been taken into consideration, since it seems likely that the production and deposition of ECM is under the control of multiple pathways [30]. In addition, recent studies have indicated that tissue fibrosis in the periurethral region of the prostate is associated with lower urinary tract symptomatology (LUTS)/the benign prostatic syndrome (BPS) in men, and have suggested that fibrosis might be an under-recognized factor in the pathobiology contributing to this disease. In response to exposure to the pro-fibrotic exogenous protein TGFβ1, prostate stromal fibroblasts can express fibrosis-associated collagen 1 and 3 and smooth muscle alpha-actin and undergo complete functional (pheno)conversion into myofibroblasts. In fact, neo- and dysplastic prostatic stromal tissue is characterized by the abundant presence of myofibroblasts. Tissue excised from the periurethral region of prostates of men with LUTS (>8 points, according to the symptom score of the American Urological Association) showed a significantly greater content of collagen and a lower glandular portion than the tissues from men without LUTS. In addition, histological proven inflammation was more pronounced in tissues from patients reporting moderate-to-severe LUTS. When combined, these findings suggest that the periurethral deposition of ECM promotes fibrotic changes in prostate tissue, and reduces urethral flexibility and compliance, thereby contributing to obstructive symptoms and the progression of LUTS [33].

## 5. Fibrosis of the Urinary Bladder

The first step in the development of fibrosis of the urinary bladder is an impairment in urinary flow to the outlet region, leading to an increase in bladder outlet resistance. Fibrotic changes contribute to the reduction in stretch flexibility, induce bladder wall stiffness and reduce bladder compliance, thus resulting in prolonged high intravesical pressure that can finally affect renal function [34]. This may cause stress to the bladder wall and induce symptoms of lower urinary tract symptomatology (LUTS), including symptoms of the overactive bladder (OAB). Later, a distension of the detrusor smooth musculature and alterations may occur in the structure of the bladder wall, such as muscular hypertrophy and increased deposition of ECM. In fact, in male adults, voiding disorders are often linked to an infravesical obstruction (for example, due to benign prostatic enlargement). It has been shown that patients diagnosed with bladder outlet obstruction (BOO) present an increased level of phosphorylation of Akt 1 which regulates the expression of compounds contributing to the induction of hypertrophy, such as type I collagen, alpha smooth muscle actin and PNCA [35]. These responses may disrupt the regulation of the growth of fibroblasts and the secretion of matrix metalloproteinases (MMPs) and tissue inhibitor metalloproteinases (TIMPs), all of which lead to the continuous deposition of ECM and accelerate the process of bladder fibrosis. In both males and females, OAB and LUT symptoms can be associated with urodynamically proven detrusor overactivity. Under normal conditions, when there is a balance between the formation and degradation of ECM, TGF β is vital in maintaining the structural integrity of the bladder, since it also plays a role in the recruitment of stem cells during the process of tissue regeneration or remodeling [36]. However, when the TGF β pathway is upregulated, for example, due to elevated oxidative stress and the production of inflammatory cytokines, and excess fibrotic tissue is formed, the smooth musculature undergoes atrophy, bladder compliance decreases and pathological changes appear [37]. In addition, external beam ionizing radiation therapy for pelvic malignancies (bladder, prostate, colorectal, endometrial or cervical cancer) might be another trigger for inflammation of the bladder mucosa, followed by edema of blood vessels, poor oxygenation and, hence, tissue necrosis. Ultimately, the differentiation of fibroblasts into myofibroblasts, and their excessive proliferation, is stimulated. The excess production of collagen and other ECM components is compounded by a reduction in the activity of enzymes that have the ability to decelerate or counteract the fibrotic remodeling. Subsequently, due to the proximity of the tissue to the area exposed to the greatest radiation, atrophy can occur to reduce compliance of the bladder and cause functional impairments (voiding dysfunction and, as a consequence, damage to the upper urinary tract) that significantly impair the quality of life of the patients. Although ureteral strictures, most often located proximal to the ureteric orifices, are serious complications of external radiation therapy, the overall incidence is very low (at <3%) [38,39].

## 6. Peyronie’s Disease

Peyronie’s Disease (PD) (also designated as Induratio penis plastica) has been named after the French surgeon François Gigot de la Peyronie (born 1678, died 1747), who contributed the first comprehensive clinical description of this particular andrological malady in 1743 [40,41]. PD is probably the fibrotic disease of the urogenital system that has been characterized and investigated most comprehensively. It is a localized disorder of the connective tissue, primarily affecting the tunica albuginea and the space between the tunica albuginea and the penile erectile tissue (corpus cavernosum). It is characterized by the development of dense, circumscribed, painless fibrous notches (plaques), resulting in an angulation of the erected penis (penile curvature). This progressive deformation can make PD a physically debilitating condition associated with variable degrees of erectile dysfunction (ED) in most cases, thus significantly affecting the quality of life of the patients and their partners. Reports indicate that from 1% up to 9% of men are affected, from the beginning of the fifth into the sixth decade of life, and the majority of cases have been reported in Caucasian men [42,43,44]. The most important factors in the pathogenesis of PD are the transformation induced by TGF β1 of primary fibroblasts of the tunica albuginea into myofibroblasts, the deposition of extracellular matrix including the stimulation of collagen synthesis and, finally, the induction of a chronic state of fibrosis [45,46]. The initial treatment of PD consists of oral or intralesional pharmacotherapy. Mainly, oral therapy with (potassium) para-aminobenzoate (POTABA^TM^, Glenwood GmbH, Munich, Germany) is most commonly employed. Clinical studies have indicated that the treatment may decrease the size of the plaques and exert a significant protective effect on the deterioration of penile curvature. Overall, para-aminobenzoate appears to be beneficial in stabilizing the disorder and preventing the progression of penile angulation [47]. Clinical studies have indicated the potential for the enzyme collagenase from clostridium histolyticum (CCH) to be used for the treatment of patients with PD. CCH comprises a heterogeneous group of seven different enzymes that digest under physiological conditions specific protein domains within type I and type III collagen fibers, the predominant collagen types found in PD plaques. It has also been shown to suppress the maturation of fibroblasts by decreasing their metabolic activity and reduce the expression of alpha-smooth muscle actin and the transforming growth factor beta 1 (TGF β1). Usually, CCH, commercially available as a mixture of AUX-I and AUX-II collagenases (XIAFLEX^TM^, Endo Pharmaceuticals Inc., Malvern, PA, USA), is applied in a total of eight intralesional injections split into four cycles of two injections (0.58 mg) given 24 h to 72 h apart from one another at intervals of six weeks. Injections of CCH are effective in patients with PD, resulting in a moderate reduction in penile curvature (by 32%), resolution of penile pain associated with PD, improvement in subjective bothersome symptoms (mean decrease 43%) and also an ability to conduct sexual intercourse. It is presumed that CCH will continue to remain an attractive modality over the following years, as a form of minimally invasive treatment of PD [48].

Since myofibroblasts are key cells in the pathogenesis of PD, the inhibition of myofibroblast transformation has been suggested as an alternative therapeutic option. It has been shown that the development of PD-like lesions is accompanied by a steady overexpression of the inducible form of the enzyme nitric oxide synthase (iNOS). By producing nitric oxide (NO) and increasing the levels of cyclic GMP, the expression of iNOS in response to the formation of fibrotic plaques may counteract fibrosis and reduce, in part, the deposition of collagen by scavenging reactive oxygen species. In a rat model of TA lesions induced by TGF ß1, the inhibition of iNOS activity by the chronic administration of L-iminoethyl-L-lysine increased the abundance of myofibroblasts and the synthesis of collagen type I. In cultures of human tissue fibroblasts from the tunica albuginea, the NO donor S-nitroso-N-acetyl penicillamine (SNAP) was seen to reduce the total number of cells positive for smooth muscle alpha actin (a marker of myofibroblasts) and to decrease the degree of immunostaining of collagen type I. This is in support of the hypothesis of antifibrotic effects of NO (produced by iNOS) [49].

As outlined above, the endogenous production of NO and subsequent increase in the levels of cyclic GMP may potentially act as an endogenous antifibrotic mechanism to reduce the deposition of collagen in the extracellular matrix. Consequently, the long-term administration of phosphodiesterase (PDE) type 5 inhibitors, known to prevent the degradation of cyclic GMP, has been suggested to counteract the development of fibrotic plaques. Indeed, by using a validated in vitro phenotypic screening assay, the PDE5 inhibitor (PDE5i) vardenafil was identified as significantly inhibiting myofibroblast transformation and the production of extracellular matrix. This was mirrored by the down-regulation of targets on both the level of mRNA and protein expression. Given the early evidence of the beneficial outcome in vitro, PDE5 inhibitors might prove to be useful in the early, non-stable phase of PD [50]. In vitro, the PDE5i vardenafil in combination with the selective estrogen receptor modulator (SERM) tamoxifen significantly inhibited, in a synergistic manner, the transformation of primary fibroblasts into myofibroblasts and the deposition of extracellular matrix. In a rat model of PD, the antifibrotic effect of the combination of vardenafil and tamoxifen was greater than that of either drug alone [51]. However, there is evidence that both PDE5 inhibitors and SERMS can only prevent to a certain degree, but not effectively or completely reverse, myofibroblast transformation and the production of extracellular matrix induced by TGF β1. In in vitro models, both compounds appear to be vital up to 36 h following treatment with TGF β1 of human primary fibroblasts isolated from the tunica albuginea. The said time interval has, thus, been considered as a “point of no return” [52]. This is in support of the view that the administration of a PDE5i or a SERM might be feasible only in the early stage of the development of PD. Since, up until today, reliable clinical data are missing, further trials using PDE5 inhibitors, either alone or in combination with other drugs (such as acetyl-L-carnitine, tamoxifen or potassium para-aminobenzoate), should be considered in order to evaluate whether these drugs might be efficacious in treating PD in the active phase.

Adenosine and adenosine receptors have also been suggested to be involved in the pathophysiological process leading to fibrosis. The adenosine receptors ADORA1 and ADORA2B were found to be expressed in both human primary fibroblasts from the tunica albuginea and cell populations derived from PD plaques. Using in-cell enzyme-linked immunosorbent assays (ICEs), it was demonstrated that the selective ADORA2B agonist BAY 60-6583 inhibited myofibroblast transformation in a concentration-dependent manner. However, taking into account the high concentrations of the ligand used in the experiments (IC_50_ was assessed to be 30 μmol), it remains to be established whether the ADORA2B receptor may hold the potential as a novel therapeutic target to treat PD during the early, non-stable phase of the disease [53]. Overall, further research will be necessary in order to identify additional targets for future concepts of pharmacotherapy in PD [54].

Alterations in the composition of the extracellular matrix of the non-vascular penile erectile smooth musculature (corpus cavernosum) in terms of the formation of collagen, fibronectin and proteoglycans and the inhibition of the proliferation of smooth muscle cells have also been suggested to play a role in the manifestation of erectile dysfunction (ED). Studies on the pathophysiological mechanisms of ED have revealed a relationship between low oxygen levels, the expression of TGF ß1, smooth muscle atrophy and the accumulation of collagen in the CC, all of which contribute to an impairment in erectile function. Tissue biopsies from the CC and plasma samples that had been taken from patients with organogenic ED revealed both a high content of collagenic fibers and an expression of TGF ß1, thus suggesting that the up-regulation of the cytokine may have an impact on the pathogenesis of ED [55,56]. The potential link between a decrease in penile blood perfusion (induced by, for example, vascular endothelial dysfunctions, diabetes mellitus or a local denervation due to the ablation of cavernous nerves brought about by injuries or pelvic surgery), a marked reduction in oxygenation (hypoxia), increased expression of TGF ß1 and the subsequent onset of cavernosal fibrosis is strongly supported by a vast number of research data [57]. Aside from erections elicited by sexual stimulation, nocturnal penile erections also contribute to achieving periodically high levels of cavernous pO_2_, thus maintaining the inhibition of collagen synthesis induced by TGF ß1 on a level sufficient to prevent the onset of penile fibrosis. The relation between a loss of nocturnal (and normal) erections, an increase in the expression of TGF ß1 and the subsequent accumulation of collagen fibers is the rationale for the early administration of PDE5 inhibitors (such as sildenafil, vardenafil or tadalafil) to patients after nerve-sparing radical prostatectomy [58,59,60,61]. This is meant to improve cavernosal blood flow and tissue oxygenation, prevent the excess deposition of extracellular matrix and support the recovery of spontaneous erections sufficiently for sexual intercourse.

## 7. Potential Future Therapeutic Strategies to Treat Genitourinary Fibrosis

Up until now, there is no medication available on the market that can cure or completely reverse fibrosis. All drugs currently used in clinical settings, such as the angiotensin II receptor antagonist losartan, para-aminobenzoate, the anti-oxidative, anti-inflammatory compound pirfenidone, and the tyrosine kinase inhibitor nintedanib, may relieve symptoms only or delay the progression of the disease. As outlined above, TGF β is a key player in the induction of fibrosis and has been found in multiple organs. The activation of TGF β induces EMT by stimulating downstream pathways, such as the activation of Smad 3 and gene expression mediated by Smad 3. Thus, it is assumed that, in the future, antagonizing the TGF β signaling pathway may provide avenues for therapeutic intervention. In particular, inhibitors of TGF β offer some advantages, and could gain significance as an effective option for controlling fibrotic conditions in the urogenital tract. Antagonizing the binding of the ligand TGF β to its heteromeric receptor complex by using a monoclonal antibody (mAB) has been reported to reduce ECM deposition and expression of fibronectin, and to attenuate fibrosis in various scenarios, including UUO and renal injury [62,63]. Therapeutic approaches for modulating the TGF β signaling cascade involve isoform-selective ABs, such as metelimumab, lerdelimumab, GC-1008 (all from Genzyme, Cambridge, MA, USA) and 2G7 (Genentech Inc., San Francisco, CA, USA), as large-molecule inhibitors targeting TGF β1 and TGF β2. Alternatively, the expression of TGF β isoforms can be affected by antisense technology targeting specific mRNA sequences. Potential drug candidates are AP-12009 and AP-11014 (developed by Antisense Pharma, Melbourne, VC, Australia). The intracellular inhibition of the TGF β RI kinase achieved by small-molecule inhibitors is another promising pharmacological approach. This group of compounds is exemplified by SB 431542 and SB 505124 (from GlaxoSmithKline, London, England, UK), and LY 580276, LY 550410, LY 573636 and LY 2157299 (all from Eli Lilly & Co., Indianapolis, IN, USA). Other inhibitors of the TGF β receptor kinase are Ki-26894 (Kirin Brewery, Nakano, Japan), SM16, A-83-01 (Kyoto Pharma/Dainippon Sumitomo Seiyaku, Kyoto, Japan) and IN-1130 (In2Gen, Irving, TX, USA). Meanwhile, dual inhibitors of both the TGF β RI and TGF β RII kinases, such as LY2109761 (Eli Lilly & Co.), have also been evaluated in preclinical settings [64,65].

As mentioned before, a number of treatment approaches to suppress or partially reverse EMT may include the application of naturally derived therapies, such as herbal compounds or compounds derived from animals. In laboratory experiments, some herbal extractions have been shown to attenuate EMT. In particular, resveratrol, a polyphenolic compound with anti-oxidative properties, extracted from the peel of red grapes, and curcumin, extracted from Curcuma longa, can partially inhibit renal EMT by regulating the Smad pathway to prevent the formation of Smad 2/Smad 3 [66,67]. Some members of the poricoic acid group, characterized as tetracyclic plant triterpenoids extracted, for example, from the mushroom Poria cocos, were found to suppress EMT by inhibiting the RAS system [68]. They were also found to competitively suppress the interaction of Smad 3 with the TGF β receptor, indicating that Smad 3 is the critical therapeutic target of the group of poricoic acids [69]. Eicosapentaenoic acid (EPA), a main constituent of fish oil, has been reported to inhibit inflammatory responses and exert anti-fibrotic activity by regulating the Smad 3 signaling and activating the expression of miR-541 [70]. To date, the function of EPA in attenuating renal EMT has only been tested in vitro, and further experiments should be carried out to confirm its efficacy. Melittin, the major component of honey bee venom, has been indicated both in vitro and in vivo to suppress EMT via interference with Smad-dependent pathways [71]. Further studies are indicated to suggest whether EPA and melittin may have the potential to alleviate fibrotic tissue transition in clinical use.

## 8. Conclusions

**Figure 1:**
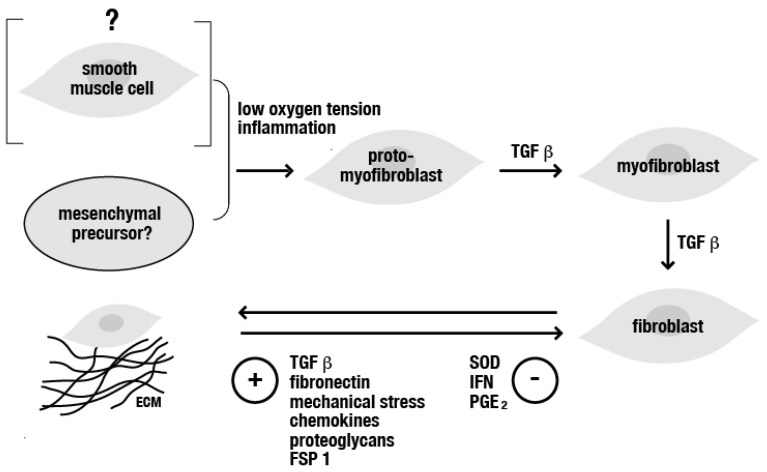
Chart displaying as to how pro-fibrotic factors (for example, inflammation and/or hypoxia) and the TGF ß (released from platelets and leukocytes) contribute to the onset of fibrosis by inducing cells, such as (proto-)fibroblasts (originating from mesenchymal precursor cells or smooth muscle cells), to produce additional TGF and collagenic fibers, finally altering the composition of the extracellular matrix and further stimulating mesenchymal transition and the accelerated deposition of ECM. Abbreviations: ECM = extracellular matrix, FSP 1 = fibroblast-specific protein 1, IFN = interferon, PGE = prostaglandin, SOD = superoxide dismutase, TGF ß = transforming growth factor beta.

Fibrotic deteriorations in tissues of the human upper and lower urinary and genital tract still remain poorly understood. This is largely due to the fact that the origin and regulation of the aberrant deposition of extracellular matrix proteins (mainly collagen type I and fibronectin) is under the control of a complex pathway involving numerous systemic inflammatory and pro-fibrotic mediators. One of these is TGF ß1, known to orchestrate the program of fibrogenesis leading to failed epithelial recovery and redifferentiation, the excess deposition of collagenic fibers, the disruption of normal vascular and neuronal innervation, and subsequently to dysfunctions of organs of the genitourinary system. Up until the present date, there is no type of medication available to completely reverse fibrosis. Clarification and better understanding of how cellular signaling pathways are activated during the process of the so-called epithelial–mesenchymal transformation (EMT), contributing to fibrotic alterations, may improve both the development of therapeutic strategies and inhibition of disease progression. In general, given the fact that fibrotic diseases of the genitourinary tract share some underlying pathological mechanisms involving a systemic pro-inflammatory state (mainly associated with disturbances in local wound healing) and increased activity of EMT, it seems likely that future research may broaden the knowledge of the pathophysiological functions of endogenous contributors to fibrotic tissue deposition and facilitate the identification of chemicals and drugs that can partially inhibit and reverse EMT, thus providing the potential to effectively tackle, and also prevent, fibrosis (Figure 1).

## Data Availability

Previously reported bibliographic data, available at PubMed/Medline, were used to support the results from this study. These prior studies are cited within the manuscript text and listed in the references.
